# Concept of Atherosclerosis Velocity: Is It a Better Measure of Cardiovascular Risk?

**Published:** 2013-09

**Authors:** Seyyed Mohammad Reza Kazemi-Bajestani, Majid Ghayour-Mobarhan

**Affiliations:** Cardiovascular Research Center, Avicenna Research Institute, Mashhad University of Medical Sciences, Mashhad, Iran

**Keywords:** Atherosclerosis, Velocity, Plaque, Risk factors

## Abstract

In most cases atherosclerosis is the underlying cause of vascular diseases, including heart disease and stroke. It is believed that endothelial injury is the earliest change in the artery wall and that this precedes the formation of lesions of atherosclerosis. Recent developments in the field of atherosclerosis have led to a renewed interest in the recognition of the parameter of time in the atherosclerosis process. We believe that the factors determining the time-dependent rate of atherosclerosis progression are important, and it is in this context that we wish to propose for the first time the term “atherosclerosis velocity”. In this review article, we summarize the existing evidence regarding atherosclerosis velocity and discuss the importance of this issue.

## Introduction

Atherosclerosis is the most important underlying cause of cardiovascular disease, a major global cause of morbidity and mortality.^1^ The prevalence of atherosclerotic cardiovascular diseases in Iran seems to be higher than that in Western countries.^2^^,^^3^ Atherosclerosis is usually characterized by the disorders of lipid metabolism, leading to low-density lipoprotein cholesterol (LDL-C) deposition in the arterial wall, which is associated with an inflammatory response and results in a plaque formation.^4^^,^^5^

It is believed that endothelial injury is the earliest change in the artery wall and that this precedes the formation of lesions of atherosclerosis. Endothelial dysfunction is associated with increased leukocyte adhesion and increased endothelial permeability to lipoproteins and other plasma constituents. This is followed by the accumulation of a mixed leukocyte population within the subendothelial space.^6^ The earliest macroscopically recognizable atherosclerotic lesions are fatty streaks**. **Lipid-laden monocytes, macrophages (foam cells), and T lymphocytes are known to be the essential components of fatty streaks.^6^ Progression to intermediate and then advanced lesions is characterized by the formation of a fibrous cap overlying a lipid-rich core. The fibrous cap is known to be a balance between the smooth muscle cells producing collagen and the macrophages degrading collagen. The thickness of the cap depends on the relative activity of those two components and there is, therefore, a danger of the fibrous cap rupturing, which may lead to acute fatal cardiovascular events.^7^

Thrombosis occurs as a consequence of a ruptured fibrous cap, and this catastrophic phenomenon is very frequent at the inflamed and thinned sites of the fibrous cap in advanced lesions. Thinning of the fibrous cap is apparently due to the continuing influx and activation of macrophages which release matrix metalloproteinases (MMPs) and other proteolytic enzymes at these sites. These enzymes cause the degradation of the matrix and can bring about thrombus formation and subsequent occlusion of the artery.^6^

**Atherosclerosis Velocity**

One important aspect of atherogenesis that we believe has not received due attention is the rate at which atherosclerosis develops. Most previous work has focused on the development and progression of atherosclerosis, but the rate of progression has been largely ignored. For example, if we ask which risk factors or a combination of which risk factors are important for the rate of atherosclerosis development, it is unclear what they may be, although accelerated atherosclerosis has been described following angioplasty or heart transplantation.^8^

We believe that the factors determining the rate of progression are important, and it is in this context that we wish to propose for the first time the term “atherosclerosis velocity”. Although the term “velocity” has not been previously employed in the context of atherosclerosis, we believe that this terminology and several aspects thereof can be drawn upon in a user-friendly way in future research. Basically, velocity is a parameter often used in physics and expresses “the rate of change of the position of an object, equivalent to a specification of its speed and direction of motion”.^9^ Velocity describes both how fast (i.e., time-dependent progression) and in what direction the object is moving. Therefore, we herein propose the term “atherosclerosis velocity” by taking into consideration plaque stability/vulnerability, which accelerates the final phase of atherosclerosis.

**Figure 1:**
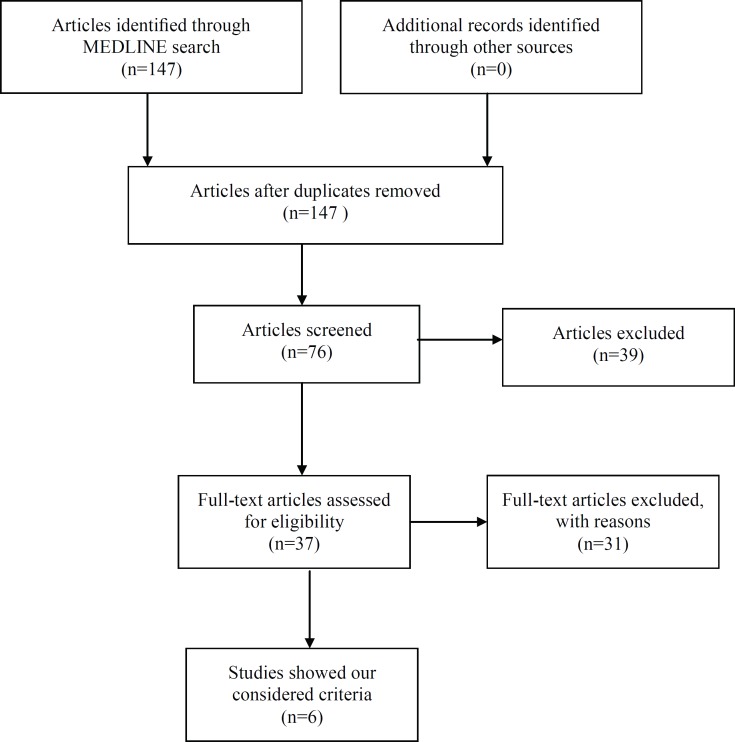
The flow chart shows the method of MEDLINE search in our article

In this review article, we summarize the existing evidence regarding atherosclerosis velocity and discuss the importance of this issue. Indeed, we seek to explore the evidence that encompasses all the three essential atherosclerosis-related factors, namely time/ duration of atherosclerosis progression, plaque volume, and plaque vulnerability, in one study. We searched MEDLINE (1970-2013) using the subsequent keywords: “atherosclerosis progression”; “vulnerable plaque”; “risk factors”; “ plaque volume”; “atherosclerosis regression”; and “atherosclerosis duration/ time”. Searches were not limited by language or study format. We found a total of 147 studies. Moreover, we screened the reference lists of the identified articles to find additional relevant publications. Overall, we considered 6 studies to be relevant to this review and summarized them (figure 1). 

The following is a description of our new terminology and discussion of some related topics.

**Phases of Atherosclerosis and Related Factors**

**Figure 2:**
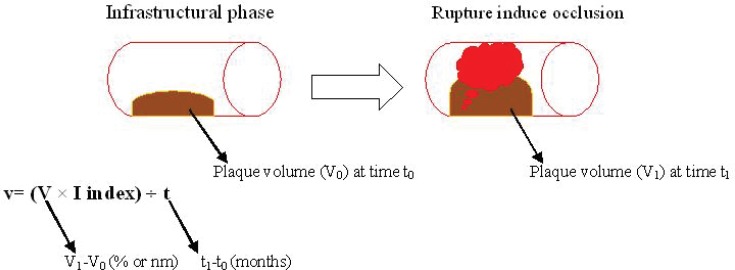
(Atherosclerosis Velocity). This figure presents the description and application of atherosclerosis velocity. This equation can be used in prospective studies for investigating the association between traditional and novel risk factors and atherosclerosis velocity

Several previous investigations have proposed different phases for atherosclerosis progression.^10^^,^^11^ We believe that atherogenesis can pragmatically be divided into two phases. The first phase covers the duration from the start of lipid deposition to subsequent plaque formation, and it may lead to stable and/or unstable plaques. This may be termed the “infrastructural” phase of atherosclerosis. In this period, the plaque may be visible or invisible (subacute) in angiography. However, new methods of imaging are capable of detecting the presence of early plaques. This first phase always happens in atherosclerosis. The second phase is frequently an acute phase, covering the duration from the point at which the plaque starts to rupture to thrombus formation, and it may give rise to acute coronary syndrome (ACS). This may be termed the “rupture-induced occlusion” phase. The second phase does not always happen in atherosclerotic patients, and subsequent ischemic events occur only because of gradual arterial narrowing in these patients (figure 2). A common finding in clinics is that the electrocardiogram (ECG) in patients with coronary artery disease, but with no previous history of myocardial infarction (MI) and also no detectable sign of MI in the ECG, shows only ischemic patterns such as T inversion or ST depression: this is a reflection of long-term ischemia without any acute infarction. After the first phase, minor ruptures and subsequent repair and also regression might occur. Nevertheless, the occurrence of a clinically relevant acute event is what constitutes the second phase. This classification is a general one that comprises all previous data regarding several phases for atherosclerosis. Furthermore, this classification is easier to use in a clinical context.

I: Index of the instability of a plaque, from +1 to +2. It either can be valued 1 for a plaque which does not experience any clinical acute event during time t or can be valued 2 for a plaque which results in acute occlusion.

V_0 (%)_ is the true percentage of lumen stenosis/ or plaque volume at t_0_ time.

V_1 (%) _is the true percentage of lumen stenosis/ or plaque volume at t_1_ time.

t (months) : t_1_-t_0_

v: atherosclerosis velocity (% or nm /months)

The pathological mechanisms leading from stable lesions to the formation of vulnerable plaques remain in doubt, and the associated clinical events are unpredictable.^12^ Several attempts have been made to use imaging techniques such as magnetic resonance imaging (MRI) to monitor the formation and progression of atherosclerotic plaques in rodents and rabbits.^12^^-^^14^

Skogsberg et al.^15^ reported that in atherosclerosis-prone mice with human-like hypercholesterolemia, atherosclerotic lesions initially progressed slowly and then showed a rapid expansion. Subsequent to advanced lesions, a plateau trend existed in these atherosclerotic mice. Accumulation of lipid-poor macrophages was demonstrated to be associated with the rapid expansion phase. 

It is important to mention that the atherosclerotic lesion is not pathologically homogeneous and atherosclerosis, far from being a linear model, is at times rapid and at others slow.^16^ The unpredictable and often episodic nature of atherosclerosis progression can be explained by the rapid increase of stenosis severity due to thrombosis.^7^

According to our proposed practical classification of atherosclerosis phases, atherosclerosis velocity includes the time-dependent development of the plaque from endothelial injury to acute arterial thrombosis. 

In terms of the phases of atherosclerosis, there is little information available on the evaluation of the factors that affect the duration of infrastructural and subsequent rupture-induced occlusion separately. If investigators focus on the concept of “time” for atherosclerosis development, it may result in considerable prevention of cardiovascular events. As a consequence, atherosclerosis-related morbidity/mortality can be effectively prevented.

**Description of Atherosclerosis Velocity**

Our suggested description of atherosclerosis velocity (v) is described in this section. It is worthy of note that this formula/description is intended, for the time being, only to further clarify the concept of atherosclerosis velocity. Accordingly, it is completely hypothetical and its application should be tested in several animal and human studies. 

v=(V×I index)÷t

V_0 _(%) is the true percentage of lumen stenosis/ or plaque volume at t_0_ time.

V_1 _(%) is the true percentage of lumen stenosis/ or plaque volume at t_1_ time.

V (%): V_1_-V_0_

t (months): t_1_-t_0_

v: atherosclerosis velocity (% or nm / months)

The I index represents the instability of a plaque and either can be valued 1 for a plaque that does not experience any clinical acute event during time t or can be valued 2 for a plaque that experiences acute occlusion/ thrombosis. 

“I” is a parameter that may change during further investigations and new items or new scoring might be added to this parameter. If new imaging methods in the future (e.g., intravascular ultrasonography, computed tomography angiography, and angioscopy) can precisely determine the “I” score at different times and preferably with a none-invasive approach, this index might become more precise and detailed.

At this point in time, the identification of the “I” score may not be very precise. The introduction of the “I” score might prove more useful when a precise method for the identification of instability is identified. However, clinical acute coronary events can always be deemed the highest “I” score. This equation can be used in prospective studies on the association between traditional and novel risk factors and atherosclerosis velocity. A summary of the description and application of atherosclerosis velocity can be observed in figure 2.

**Importance of Atherosclerosis Velocity**

We believe that in several previous investigations, atherosclerosis velocity has not been sufficiently studied. In other words, as much as we currently know various parameters believed to be the causative or consequence factors of atherosclerosis, we really do not have a good understanding of the effects of these factors on atherosclerosis velocity. 

Saremi et al.^17^ reported that Pioglitazone, a drug of the Thiazolidinedione class with hypoglycemic action to treat diabetes, slowed the progression of carotid intima media thickness (IMT) during an average follow-up of 2.3 years compared to placebo. Imagine if another study examines compound X in a matched group of patients and reaches the same curve of IMT decrease but in one year; it would mean that compound X could decrease atherosclerosis velocity almost by 50%. Another example in this regard is the study of Yamazaki et al.^18^ They showed that in patients under statin therapy at a 12-month measurement point, mean-IMT change was correlated with LDL-C and LDL-C/ HDL-C.

Sun et al.^19^ recently performed an interesting study which almost combined all three parameters of time/ duration, plaque volume, and plaque vulnerability/instability characteristics. The authors characterized the impact of atherosclerosis on the short-term (6 months) natural history of the lipid-rich necrotic core (LRNC) in carotid artery plaques using MRI and concluded that LRNC was essentially affected by the characteristics of plaque stability, which seemed to be even more important than clinical features.

Several previous articles have concluded that atherosclerosis is a chronic disease.^4^^,^^5^ However, we think that it is time we considered the term “acute atherosclerosis”. Acute atherosclerosis represents a rupture-induced occlusion and is a disorder that may develop even a very short time after plaque formation. 

Atherosclerosis velocity has dependency on plaque stabilization and acute rupture. Therefore, if we assume that the endpoint of atherosclerosis is acute coronary occlusion and/or gradual arterial narrowing-induced ischemia, we should then turn our attention to the risk factors that contribute to a rise in atherosclerosis velocity. Inflammation is known to be a crucial component of atherosclerosis^10^^,^^20^^,^^21^ and plays an important role in plaque instability.^22^ Indeed, time and plaque volume are also two important factors in atherosclerosis development and progression. Be that as it may, from a clinical perspective, a combination of all the three parameters of plaque volume, time of plaque progression, and instability indices of plaques is critical.

Atherosclerosis velocity may show a wide range in future studies. When a small unstable plaque can rapidly rupture and result in total coronary occlusion and when a large plaque can persist for a longer time (or at least when it does not lead to complete occlusion), we can easily see the importance of atherosclerosis velocity. Clinically, atherosclerosis velocity vis-à-vis an asymptomatic/sub-acute arterial plaque is a highly unpredictable process. Asymptomatic/sub-acute vulnerable plaques in coronary arteries account for a significant level of acute cardiovascular events.^23^ Their main risk is associated with their acute rupture, which may result in fatal MI or stroke.^23^ Recently, the role of microcalcifications embedded in the vulnerable fibrous cap in the development of acute ruptures has been highlighted.^24^^,^^25^ Liang et al.^26^ performed an interesting study using intravascular ultrasound (IVUS) in patients and proved that the occurrence of a microcalcification in the atherosclerotic plaque fibrous cap considerably increased the risk of the rupture of a vulnerable plaque. IVUS also seems to be capable of quantifying atherosclerotic plaques as well as positive and negative vascular remodeling.^27^ Intraplaque hemorrhage also has been considered a factor which accelerates sub-clinical atherosclerosis.^28^^-^^30^

**Risk Factors and Atherosclerosis Velocity**

Regarding atherosclerosis velocity, we believe that we should be extremely precise when indicating the impact of risk factors on all the elements of atherosclerosis velocity. To our knowledge, there is currently a lack of evidence in terms of the effects of traditional cardiovascular risk factors (hypertension, hyperlipidemia, diabetes mellitus, and smoking) on atherosclerosis velocity. The effects of these traditional risk factors have been proved in atherosclerosis development and progression.^31^^-^^33^ Regardless of the effects of these traditional risk factors on the development of the atherosclerotic plaque, a growing body of evidence demonstrates their impact on rupture-induced occlusion. 

Mauriello et al.^34^ analyzed a large number of endarterectomy specimens from symptomatic and asymptomatic patients to explore the association between cardiovascular risk factors and carotid plaque morphology. The authors succeeded in proving a strong association between hypertension and vulnerable and thrombotic carotid plaques.^34^ Diabetes mellitus/hyperglycemia-induced oxidative stress/reactive oxygen species is one of the factors that can promote both vascular smooth muscle cell proliferation/migration in atherosclerotic lesions and vascular smooth muscle cell apoptosis, which results in atherosclerotic plaque instability and rupture.^35^ Macrophages, which seem to be crucial components of unstable plaques, play an important role in the destabilization process, whereas smooth muscle cells contribute to plaque stability.^36^ Several attempts have been made to propose novel techniques for the detection of macrophage-rich atherosclerotic plaques in hyperlipidemic animals.^37^^,^^38^

Other putative and novel risk factors like increased inflammatory response-related factors (e.g., C-reactive protein [CRP]) also have been shown to be effective in atherosclerosis development.^39^^-^^41^ Variation in trace elements also plays a crucial role in the initiation and establishment of atherosclerosis.^42^^-^^44^ The effects of these putative and novel risk factors on atherosclerosis velocity also have not been revealed in previous studies. Risk factors still cannot predict cardiovascular events perfectly insofar as atherogenesis is a multi-step process and critical transitions between the aforementioned phases of atherosclerosis require a complex of risk factors, which may differ for each step.^45^

**Imaging and Biochemical Biomarkers: a Key for Further Atherosclerosis Velocity Studies**

It has been demonstrated that an inflamed arterial wall with upregulated adhesion molecules is a basic factor which leads to leukocyte migration into the arterial wall; and with increasing levels of activated leukocyte products (like interleukin 6), hepatic CRP may be induced.^39^ Inflammation has been shown to be allied to the presence and severity of atherosclerotic vascular disease.^46^

Deposition of LDL-C over the inflamed arterial wall results in fatty streak formation by recruiting vascular smooth muscle cells and can eventually form fibrous plaques.^39^ Fibrous plaques are the end product of the infrastructural phase of atherosclerosis. Due to the characteristics of the fibrous plaque (stable or unstable), the subsequent second phase of atherosclerosis is expected.^47^^,^^48^

Several invasive and noninvasive techniques have been proposed to assess the quality of atherosclerotic plaques. Optical coherence tomography (OCT) and IVUS have shown sufficient feasibility to characterize lipid-rich plaques and fibrous plaques.^49^^,^^50^ As regards the volume of plaques, MRI seems to be a reliable noninvasive technique for tracking the regression and progression of atherosclerotic plaques.^30^ Recently, a combination of multi-vessel IVUS and near-infrared spectroscopy techniques has exhibited promising efficacy in the detection of the development of inflamed fibroatheromas with thinner fibrous caps, greater plaques, and necrotic core areas possessing the characteristics of increased plaque instability.^51^

We think that one of the most important factors which determine atherosclerosis velocity is the mechanical stability of the plaque. Unstable plaques with thinner fibrous caps and an excess of inflammatory cells in the outer region^47^^,^^52^^,^^53^ are prone to induce acute thrombosis and subsequent cardiac events. However, all ruptures may not result in ACS.^54^ Several assumptions inherent in the usefulness of biochemical biomarkers may not be entirely valid in the prediction of acute events/plaque rupture.^55^ The emerging application of nanotechnology for the diagnosis and management of vulnerable atherosclerotic plaques seems to be promising for future studies.^56^

At present, we do not have any accurate biomarkers for the instability index.^57^ Nonetheless, several biomarkers have previously proved relatively efficient in the prediction of plaque instability (e.g., CRP, MMPs, and heat shock proteins).^58^^-^^60^ Recently, molecular imaging of atherosclerosis has demonstrated acceptable efficacy in animal studies, but such methods have yet to be fully explored in human studies.^10^

**Plaque Regression: Atherosclerosis Velocity Slowdown**

In regard to plaque regression, time-dependent regression is also of significance (i.e., slowing down atherosclerosis velocity). We think that we should focus on the factors which exacerbate atherosclerosis velocity in order to be able to prevent ACS. Risk factor modification is a tool which may decrease atherosclerosis velocity by preventing plaque volume growth, decreasing the duration of atherosclerosis progression, and thwarting factors which may result in plaque instability (e.g., smoking cessation). Tani et al.^61^ conducted a 6-month prospective observational study on 114 patients with coronary artery disease using volumetric IVUS to asses the atherosclerosis plaque volume. They concluded that a change in the LDL-C/HDL-C ratio was a clinical tool for the prediction of plaque volume regression. This interesting study characterized an important factor which reduces atherosclerosis velocity and consequent plaque volume regression. 

High-density lipoprotein cholesterol (HDL-C) is thought to be involved in reverse cholesterol transport.^62^ Also, HDL-C has antioxidant properties and may attenuate the impact of oxidative stress on LDL-C.^16^^,^^63^ Therefore, high levels of HDL-C are associated with a reduction in the development of atherosclerotic cardiovascular diseases through the accumulation of too much cholesterol.^64^ Data from the Framingham Study suggest that a 0.03 mmol/L increase in HDL-C levels is associated with a 3% decrease in the incidence of coronary artery disease in women compared with a 2% decrease in men.^65^ Feig et al.^66^ stated that HDL-C promoted rapid atherosclerosis regression in mice and altered the inflammatory properties of plaque monocyte-derived cells. It seems that HDL-C improvement has a crucial role in the reduction of atherosclerosis velocity.

Statins are known to be capable of regressing atherosclerotic plaques.^67^^,^^68^ Nevertheless, the effects of statins, specifically on atherosclerosis velocity, are not clear. Two important meta-analyses suggest that statin therapy results in atherosclerosis regression when LDL-C is substantially reduced and HDL-C is increased.^69^^,^^70^ Statins are believed to be effective in the reduction of pro-oxidant/antioxidant balance as well as inflammation-induced atherosclerosis progression.^71^^,^^72^

Antiplatelet therapy seems to be effective in reducing atherosclerosis velocity by inhibiting both the first and second phases of atherosclerosis.^73^ Anti-inflammatory effects of antiplatelet medication are effectual in atherosclerosis velocity reduction by decreasing the volume of atherosclerosis plaques.^73^ Also, antiplatelet therapy through inhibiting the adverse effects of activated platelets can indirectly raise the stability status of plaques^73^ and subsequently lessen atherosclerosis velocity. Decreased inflammatory process in atherosclerosis plaques also directly leads to increased plaque stability.^16^ However, the effect of time-related reduction on antiplatelets should be clarified in future studies. 

All previous investigations have focused only on the probability of plaque regression at the expense of almost neglecting the imperative parameter of time. We recommend that future studies be designed based on the probable association between statin therapy and atherosclerosis velocity reduction. 

## Conclusion

We proposed a new concept in the field of atherosclerosis by suggesting the term “atherosclerosis velocity”, which encompasses all the three essential parameters of volume of plaque, time/ duration of plaque progression, and/or acute rupture and plaque stability. Our review article reveals that the previous studies have not sufficiently probed into these three parameters. We believe if the concept of atherosclerosis velocity is applied in further experiments, especially in experimental models, we can expect a practical curve of atherosclerosis. 
